# Histone acetyltransferase HAT4 modulates navigation across G2/M and re-entry into G1 in *Leishmania donovani*

**DOI:** 10.1038/srep27510

**Published:** 2016-06-08

**Authors:** Aarti Yadav, Udita Chandra, Swati Saha

**Affiliations:** 1 Department of Microbiology, University of Delhi South Campus, New Delhi-110021, India

## Abstract

Histone acetyltransferases impact multiple processes. This study investigates the role of histone acetyltransferase HAT4 in *Leishmania donovani*. Though HAT4 was dispensable for survival, its elimination decreased cell viability and caused cell cycle defects, with HAT4-nulls experiencing an unusually long G2/M. Survival of HAT4-nulls in macrophages was also substantially compromised. DNA microarray analysis revealed that HAT4 modestly regulated the expression of only a select number of genes, thus not being a major modulator of global gene expression. Significantly, *cdc20* was among the downregulated genes. To ascertain if decreased expression of *cdc20* was responsible for HAT4-null growth and cell cycle defects we expressed LdCdc20 ectopically in HAT4-nulls. We found this to alleviate the aberrant growth and cell cycle progression patterns displayed by HAT4-nulls, with cells navigating G2/M phase and re-entering G1 phase smoothly. HAT4-nulls expressing LdCdc20 ectopically showed survival rates comparable to wild type within macrophages, suggesting that G2/M defects were responsible for poor survival of HAT4-nulls within host cells also. These are the first data analyzing the *in vivo* functional role of HAT4 in any trypanosomatid. Our results directly demonstrate for the first time a role for Cdc20 in regulating trypanosomatid G2/M events, opening avenues for further research in this area.

Leishmaniases are a group of diseases currently endemic to 98 countries. According to WHO 12 million people are afflicted by Leishmaniases, with 1.3 million new cases each year. This group of diseases mainly affects people living in poverty, with malnutrition and poor housing/sanitation being major contributory factors. There are three forms of the disease - cutaneous, sub-cutaneous and visceral, with visceral leishmaniasis (VL) being fatal if not treated in timely manner. Every year 200,000 to 400,000 new cases of VL are reported, and 90% of these cases occur in Brazil, the Indian subcontinent, Sudan and Ethiopia. While there are drugs to treat VL, due to emerging drug resistance the search for new sites for therapeutic intervention continues, underlining the importance of working towards a better understanding of the pathogen’s cellular processes.

Eukaryotic DNA is organized into chromatin, the basic unit of which is the nucleosome. Barring the unstructured N-terminal tails, the core histone proteins of the nucleosome are globular in nature. Histones carry a large number of post-translational modifications (PTMs), especially on their N-terminal tails, with at least eight different types of modifications having been unearthed so far^1^,^2^,^3^. The wide range of histone PTMs bestows chromatin with the potential to regulate a variety of cellular processes such as DNA replication, repair, recombination and transcription. The structure of chromatin is coordinately modulated by chromatin remodeling complexes and histone modifications during these various DNA-related transactions. Histone PTMs are widely conserved across eukaryotes from yeast to mammalian cells, and the functional roles of these conserved modifications are also mostly conserved. Trypanosomatid histones, however, are divergent in sequence from those of other eukaryotes, and thus the PTMs they carry also vary.

While the assortment of PTMs carried by *Leishmania* histones have not directly been identified, as their sequence is highly conserved with the sequence of *Trypanosoma* histones it is likely that histones of *Leishmania* species carry the same PTMs as the histones of *Trypanosoma* species. Histone PTMs have been identified in *Trypanosoma brucei* and *Trypanosoma cruzi* by mass spectrometry analysis of the isolated histones^4^,^5^,^6^. Mature core histones lack the initial methionine residue, with the N-termini of H2A, H2B and H4 carrying a novel histone PTM, mono-methylated alanine (me-1 Ala). While the N-termini of H2A and H2B have hardly any modifications, the C-terminus of H2A is extensively acetylated, a feature that is peculiar to trypanosomes. H3 carries modifications on its N-terminal tail, but H3K4 methylation is the only specific modification that has been identified^7^. Contrastingly, several acetylation (and methylation) marks have been identified on the H4 N-terminal tail. The sites of H4 acetylation include H4K2, H4K4, H4K5, H4K10 and H4K14. Enhanced acetylations of the N-terminal tails of histones H3 and H4 have been detected in the strand switch regions of the divergent polycistronic gene arrays of *Trypanosoma cruzi*^7^ and *Leishmania major*^8^.

The MYST-family of histone acetyltransferases (HATs) get their name from the first members of this family to be identified – **M**OZ, **Y**bf2/Sas3, **S**as2 and **T**IP60, and the members of this family are characterized by the presence of an acetyltransferase domain that harbours the catalytic center of the acetyl group transfer reaction^9^. MYST-family HATs have a bearing on replication, transcription, recombination and repair, and often function as part of multi-protein complexes *in vivo*. Four MYST family HATs have been annotated in *Leishmania* species^10^,^11^, and previous work from our laboratory has found that *Leishmania donovani* HAT3 (LdHAT3) specifically acetylates H4K4 (both *in vitro* and *in vivo*), while *Leishmania donovani* HAT4 (LdHAT4) specifically acetylates H4K14 *in vitro*^12^,^13^. We found H4K4 acetylation to play a role in histone deposition, and LdHAT3 plays a role in modulating the cell’s response to UV-induced DNA damage^13^. The data presented in the following study represent the results of our efforts to investigate the role of LdHAT4 in modulating *Leishmania* cellular events. Our results implicate a role for HAT4 in regulating *Leishmania* cell cycle progression, particularly the cell’s navigation through G2/M phase. The molecular basis of this regulation has been identified.

## Results

### Creation of HAT4 knockout line

**Figure 1:**
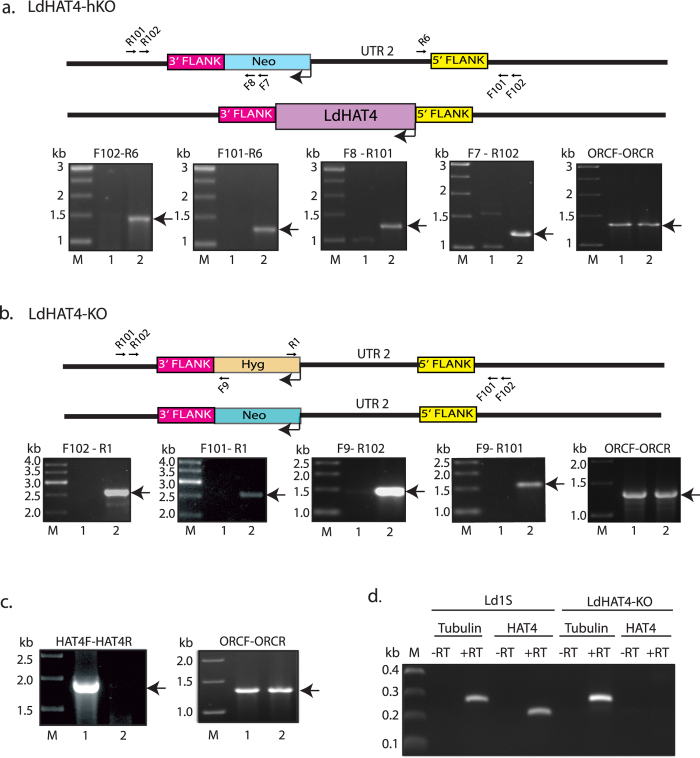
Creation of HAT4 knockout. (**a**) Screening of heterozygous knockout line (LdHAT4-hKO) by PCRs. Primers were designed using sequences of the *neo*^*r*^ cassette, UTR2, and HAT4 flanks in the *Leishmania* genome. Primer positions are marked in the line diagram. Primer pair used in each PCR is indicated above the agarose gel image. OrcF - OrcR PCR: positive control for templates. Lanes 1- Ld1S genomic DNA, lanes 2- LdHAT4-hKO genomic DNA, lanes M- DNA ladder. (**b)** Screening of knockout line (LdHAT4-KO) by PCRs. Primers were designed using sequences of the *hyg*^*r*^ cassette and HAT4 flanks in the *Leishmania* genome; positions of primers are marked in the line diagram; primer pair used in each PCR is indicated above gel image. Lanes 1- Ld1S genomic DNA, lanes 2- LdHAT4-KO genomic DNA, lanes M- DNA ladder. (**c)** Confirmation of HAT4 deletion in LdHAT4-KO line. PCRs carried out using HAT4 primers, with Ld1S genomic DNA (lane 1) and LdHAT4-KO genomic DNA (lane 2) as templates. (**d)** Analysis of HAT4 transcription in Ld1S and LdHAT4-KO cells. –RT: non-reverse transcribed RNA as template (control for checking genomic DNA contamination). +RT: reverse transcribed RNA as template. Positive control: reactions with tubulin primers.

Of the four MYST-family HATs identified by annotation of *Leishmania* whole genome sequence, three (HATs 1–3) are conserved in *Trypanosoma* species as well. HAT4, however, though present in *T*. *cruzi*, is absent in *T. brucei*. Previous work from our laboratory has found *Leishmania donovani* HAT4 (LdHAT4) to behave somewhat differently from HATs 1–3, being predominantly cytosolic in nature unlike the constitutively nuclear HATs 1–3 12. LdHAT4 was found to specifically acetylate histone H4 at K14 *in vitro*. While no information on the *in vivo* status of H4K14 acetylation is available across *Leishmania* species this histone PTM has been identified in *Trypanosoma* species, though less than 1% of H4 is reported acetylated at this position^4^,^6^. To address the role of LdHAT4 in modulating *Leishmania* cellular events we created a HAT4-null line, by sequentially replacing the two genomic alleles with neomycin resistance and hygromycin resistance cassettes respectively, as described in Methods. Clonal lines of the HAT4 heterozygous knockout line (LdHAT4-hKO) created in the first step by replacement of one allele with the neomycin resistance gene, as well as of the HAT4-null line (LdHAT4-KO) created from it by subsequent replacement of the second allele with the hygromycin resistance gene, were screened for pristine homologous recombination at both ends by PCRs across the deletion junctions using their genomic DNA as template. The primers used in these PCRs were designed against the drug resistance cassettes and the genome sequences flanking the HAT4 gene that lie beyond the donor boundaries, and both, LdHAT4-hKO as well as LdHAT4-KO lines were verified as authentic recombinants (Fig. 1a,b respectively). The absence of the HAT4 gene in LdHAT4-KO cells was confirmed in PCRs using HAT4-specific primers (Fig. 1c), and results of RT-PCR using RNA isolated from LdHAT4-KO cells corroborated the fact that HAT4 was not being expressed in LdHAT4-KO cells (Fig. 1d).

### HAT4-nulls show decreased cell viability and aberrant cell cycle progression pattern

**Figure 2:**
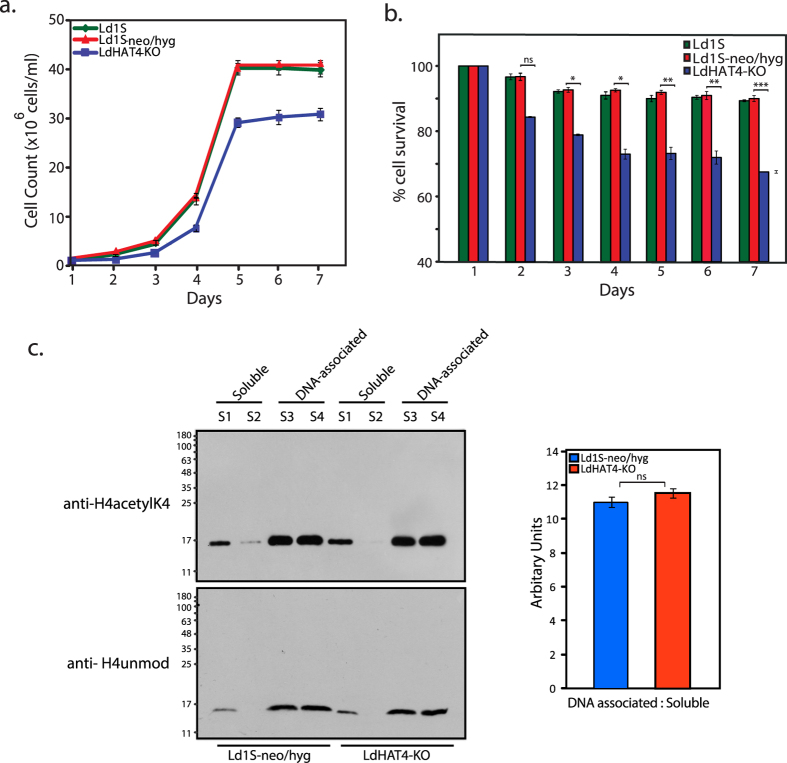
(**a)** Analysis of growth. Growth pattern of LdHAT4-KO cells was compared with those of wild type Ld1S and Ld1S-neo/hyg control lines. (**b**) Analysis of cell survival. Percent LdHAT4-KO survivors was compared with percent survivors in control lines, every 24 hrs over a period of seven days. For growth and cell survival analyses, three biological replicates were initiated and carried out in parallel, and mean values are presented, with error bars indicating standard deviation. Student’s t-test (two-tailed) was applied to analyze the data. *P* values obtained: ns_=_non-significant, *p < 0.05, **p < 0.005, ***p < 0.0005. (**c)** Analysis of DNA-associated and soluble protein fractions. Left panels: S1 to S4 lysate fractions isolated from logarithmically growing cells were resolved by SDS-PAGE (5 × 10^6^ cell equivalents per cell type) and analyzed by western blot with anti-unmodified H4 antibodies (1:10000 dilution^13^) and anti-H4acetylK4 antibodies (1:1000 dilution^13^). S1, S2: soluble fractions. S3, S4: DNA-associated fractions. Right panel: ratio of DNA associated H4 (modified + unmodified) : soluble H4 (modified + unmodified) in Ld1S-neo/hyg and HAT4-KO cells, as determined by quantification using Image J.

To analyze the impact of HAT4 deletion on cell viability the growth pattern of LdHAT4-KO cells was compared with wild type cells (Ld1S) or Ld1S control line expressing both neomycin and hygromycin resistance cassettes (Ld1S-neo/hyg^13^, and it was found that LdHAT4-KO cells showed decreased growth, with the number of viable LdHAT4-KO cells being ~75–80% the number of viable Ld1S or Ld1S-neo/hyg cells at stationary phase (Fig. 2a). To ascertain if this was the result of cell death or increase in generation time, the number of live and dead cells were scored over the week. Greater cell death was observed in case of LdHAT4-KO cells as compared to wild type (~70% cell survival in case of LdHAT4-KO cells as compared to ~90% cell survival in case of wild type cells at 7 days; Fig. 2b). Previous work from our laboratory showed that deletion of LdHAT3 led to decreased cell viability due to defects in histone deposition^13^. To examine the possibility of a similar mechanism being in play in HAT4-nulls we compared the distribution of histone H4 between soluble and chromatin-bound fractions, in HAT4-nulls versus wild type cells. Unlike HAT3-nulls where about 50% of H4 was in the soluble fraction, the distribution pattern of H4 in HAT4-nulls was found to be similar to that seen in wild type cells, with H4 being predominantly chromatin-bound (Fig. 2c). This suggests that decreased viability of HAT4-nulls is not due to defective histone deposition. We then examined the possibility of cell cycle-related defects lowering cell viability of HAT4-nulls.

**Figure 3:**
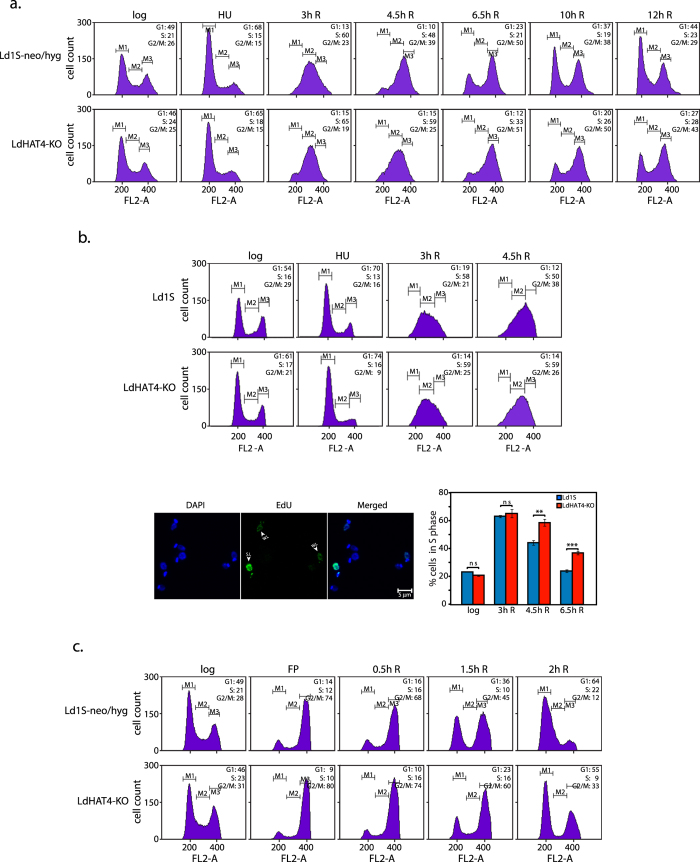
Comparison of flow cytometry profiles of LdHAT4-KO cells with those of Ld1S-neo/hyg cells. (**a**) Promastigotes synchronized at G1/S transition using 5 mM hydroxyurea and then released into S phase. (**b**) Promastigotes synchronized using hydroxyurea and then released into S phase. Aliquots of cells were pulsed with EdU for 15 min at 3 h, 4.5 h and 6.5 h after release. Upper panels: flow cytometry analysis at different time intervals. Bottom left panels: representative field showing EdU-labeled and unlabeled cells. Bottom right panel: bar chart representing percent cells in S phase at each time-point (corresponds to percent EdU-labeled cells). Pulsing of cells with EdU at each time-point was set up in triplicate. Approximately 80–120 cells were analyzed from each technical replicate, and values presented in the bar chart are mean values, with error bars indicating standard deviation. Student’s t-test (two-tailed) was applied to analyze the data. *P* values obtained: ns_=_non-significant, **p < 0.005, ***p < 0.0005. (**c**) Promastigotes synchronized at G2/M using 5 μM flavopiridol and then released into G1 phase. In all flow cytometry panels the time indicated above each histogram indicates the time after release from block (eg 3h R indicates 3 hours after release). The synchronization regime experiments in (**a**) and (****) were each performed three times as detailed in Methods, and one dataset for each regime is shown here. For each cell type 30,000 events were recorded at every time-point, and data were analyzed using CellQuest Pro Software (BD Biosciences). M1, M2 and M3 gates indicate G1, S and G2/M phases respectively. Numbers at upper right in each panel represent percent cells at each cell cycle stage. Combined statistical analyses of all data sets are presented in Supplementary Tables 1 and 2.

The effect of HAT4 deletion on cell cycle progression was examined by arresting LdHAT4-KO promastigotes at the G1/S boundary using hydroxyurea and then releasing the promastigotes into S phase synchronously, as described in Methods. Cells were harvested at various time-points after release into S phase, and analyzed for their DNA content using flow cytometry (Fig. 3a). We observed that HAT4-nulls entered and reached mid-S phase in a timely manner comparable with Ld1S-neo/hyg cells (3 h R time-point); however, they took longer than usual to navigate the remaining segment of S phase (4.5 h R time-point), and remained arrested at G2/M phase for a prolonged period, gradually returning to G1 much after Ld1S neo/hyg cells (Fig. 3a and Supplementary Table S1).

We then examined DNA synthesis in synchronized promastigotes at different time intervals following release from hydroxyurea, by pulse-labelling cells with EdU and analyzing the cells subsequently by confocal microscopy (detailed in Methods), simultaneously also sampling cells for flow cytometry analysis. Cells were counted and placed in three categories – unlabeled with EdU, weakly labeled with EdU and strongly labeled with EdU. The numbers of weakly labeled and strongly labelled cells were added up to determine the number of cells whose DNA was replicating (cells in S phase) at each time-point. As seen in the bar chart in Fig. 3b, the number of cells in S phase (corresponding to EdU-labelled cells, i.e. cells undergoing DNA replication) at the 3 hR time-point was comparable between HAT4-nulls and Ld1S-neo/hyg cells; however, on comparing the number of S phase cells in the later time-points it appears that the latter portion of S phase is somewhat prolonged in HAT4-nulls. These results suggest that LdHAT4 controls events related to DNA replication, and are in keeping with findings in *T. cruzi* where H4K14 acetylation has been found to regulate both, transcription and replication^14^.

To confirm that HAT4 deletion negatively impacts the cell’s transition across G2/M and re-entry into G1 phase, LdHAT4-KO cells were arrested at G2/M using flavopiridol (as described in Methods) and released into complete drug-free medium. A delayed release into G1 was observed in case of LdHAT4-KO cells as compared to Ld1S-neo/hyg cells (Fig. 3c and Supplementary Table S2), reinforcing our conclusion that HAT4-nulls experience a longer than usual G2/M phase.

### Cell viability defects and aberrant cell cycle progression pattern of HAT4-nulls are alleviated by ectopic expression of HAT4

**Figure 4:**
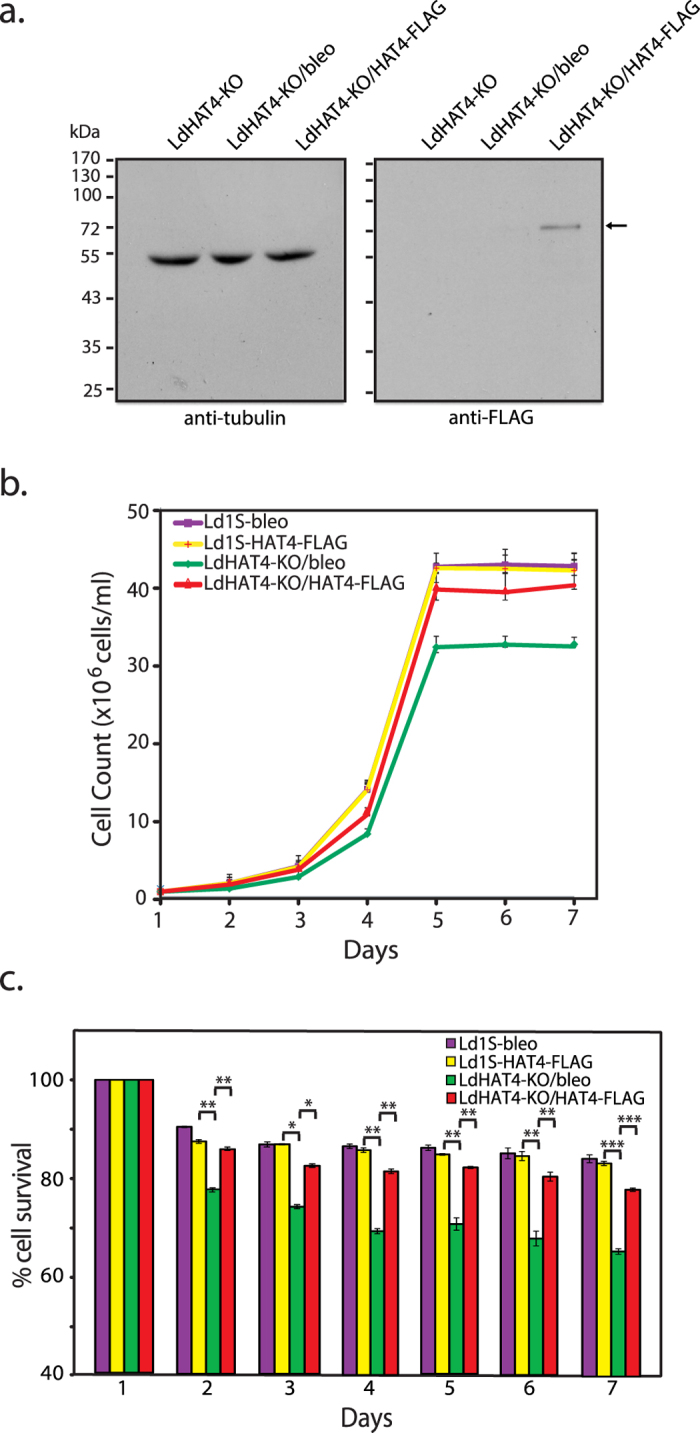
(**a**) Analysis of ectopic expression of HAT4-FLAG in rescue line. Western blot analysis of whole cell lysates isolated from LdHAT4-KO cells, LdHAT4-KO cells harbouring empty vector (LdHAT4-KO/bleo), and LdHAT4-KO cells expressing HAT4-FLAG (LdHAT4-KO/HAT4-FLAG). Anti-FLAG antibody (Sigma Aldrich) and anti-tubulin antibody (Zymed, USA) were used at 1:1000 and 1:5000 dilution respectively. **(b**) Growth analysis. Growth pattern of the rescue line (LdHAT4-KO/HAT4-FLAG) was compared with that of knockout line harbouring empty vector (LdHAT4-KO/bleo), and with Ld1S either harbouring empty vector (Ld1S/bleo) or expressing HAT4-FLAG episomally (Ld1S/HAT4-FLAG). (**c)** Analysis of cell survival. Percent LdHAT4-KO/HAT4-FLAG survivors was compared with percent survivors in knockout line LdHAT4-KO, as well as Ld1S/bleo and Ld1S/HAT4-FLAG lines, every 24 hrs over a period of seven days. For growth and survival analyses, three biological replicates were initiated and carried out in parallel, and mean values are presented, with error bars indicating standard deviation. Student’s t-test (two-tailed) was applied to analyze the data. *P* values obtained: *p < 0.05, **p < 0.005, ***p < 0.0005.

**Figure 5:**
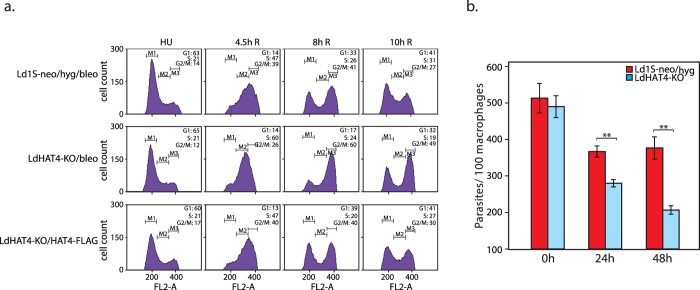
(**a**) Flow cytometry profile of rescue line in comparison with control and HAT4 knockout lines. Promastigotes of Ld1S-neo/hyg/bleo, LdHAT4-KO/bleo and LdHAT4-KO/HAT4-FLAG were arrested at G1/S using 5 mM hydroxyurea and then released into S phase. Time indicated above each histogram indicates the time after release from block. The experiment was carried out three times as detailed in Methods and one dataset is presented here. For each cell type 30,000 events were recorded at every time-point, and data were analyzed using CellQuest Pro Software (BD Biosciences). M1, M2 and M3 gates indicate G1, S and G2/M phases respectively. Percent of cells in G1, S and G2/M at the different time-points are indicated in the upper right-hand corner of each histogram box. (**b)** Analysis of survival of HAT4-null parasites within macrophages. The number of parasites within macrophages were scored microscopically using DAPI staining and Z-stack image analysis with the help of a confocal microscope. Three replicates of the experiment were separately initiated and carried out in parallel. Mean values are presented in the bar chart. Error bars indicate standard deviation. Student’s t-test was applied to analyze the data. *P* values obtained: **p < 0.005.

Attempts were made to rescue the HAT4-null mutant phenotype by expressing HAT4 ectopically in HAT4-nulls. For this, plasmid pXG(*bleo*)/HAT4-FLAG was transfected into LdHAT4-KO cells and clonal lines assessed for expression of LdHAT4-FLAG, using Western blot analysis with anti-FLAG antibodies. A line displaying robust LdHAT4-FLAG expression (Fig. 4a) was analyzed further. Cell growth and survival was monitored over seven days, and it was found that the growth and survival defects exhibited by LdHAT4-KO cells were largely alleviated in HAT4-KO cells expressing HAT4-FLAG (LdHAT4-KO/HAT4-FLAG cells; Fig. 4b,c). Ectopic expression of HAT4-FLAG in wild type cells did not have any impact on growth kinetics (Ld1S/HAT4-FLAG cells). When the cell cycle profile of the rescue line was compared with that of HAT4-null line harbouring empty vector (LdHAT4-KO/bleo cells) in hydroxyurea-synchronized promastigotes, it was observed that the prolonged G2/M phase evident in LdHAT4-KO/bleo cells was relieved in LdHAT4-KO/HAT4-FLAG cells, with cells re-entering G1 phase much faster (Fig. 5a).

### HAT4-nulls exhibit decreased survival rates in macrophages

*Leishmania* parasites exist as flagellate promastigotes in the insect host, initially as non-infective procyclic forms in the midgut and finally as infective metacyclic forms in the salivary glands, which get released into the bloodstream of the mammalian host upon insect bite. These infective metacyclics are taken up by macrophages where they evade the host’s defence systems, differentiate into amastigotes, and reproduce by binary fission. Eventually the amastigotes are released from the macrophages, whereupon they infect other macrophages and the cycle continues. A similar pattern of development is observed in axenic cultures. To examine the effect of HAT4 deletion on survival of the parasite within host macrophages, J774A.1 cells were infected with *Leishmania* metacyclics (either Ld1S-neo/hyg or HAT4-nulls) as described in Methods. Parasite infections were analyzed by acquiring Z-stack images of infected macrophages using confocal microscopy. The number of parasites within infected macrophages were counted right after the five hour incubation of macrophages with metacyclics was complete (0 h time-point), and 24 hrs and 48 hrs after the 0 h time-point. We observed comparable infectivity and parasite load of HAT4-nulls and control parasites at 0 hrs (Fig. 5b). This indicated that uptake of parasites by macrophages was unaffected by deletion of HAT4 in *Leishmania* promastigotes, underlining the fact that differentiation into metacyclics was unaffected by HAT4 deletion in promastigotes. The number of HAT4-null parasites was ~75% the number of Ld1S-neo/hyg parasites per hundred macrophages 24 hours later, and a sharper difference in the number of parasites per hundred macrophages was detected 48 hours later, with HAT4-null parasites numbering ~ 40% the number of control parasites (Fig. 5b). Thus, HAT4 appears to be important for parasite growth within macrophages also.

### LdHAT4 modestly regulates expression of a select group of genes

**Figure 6:**
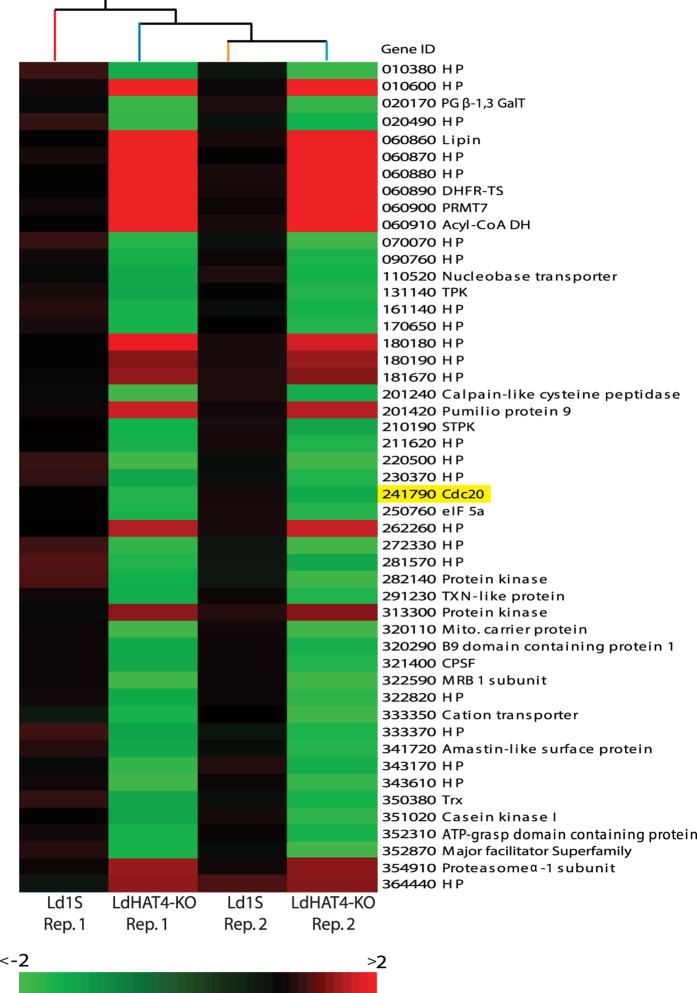
DNA microarray analysis of HAT4-nulls in comparison with wild type cells. Microarray analysis was carried out using the Agilent–based platform GPL18125, as described in Methods. Biological replicates of each cell type were analyzed, and comparative analyses of gene expression in LdHAT4-KO cells with reference to wild type cells was carried out using GeneSpring GX Version 12.1. The heat map presented here shows genes that were differentially expressed in LdHAT4-KO cells as compared to control Ld1S, with green signifying downregulated genes and red signifying upregulated genes. Rep.1: replicate 1, Rep.2: replicate 2. Complete data available at Gene Expression Omnibus, Accession GSE76574.

Histone acetylation has long been known to positively regulate transcriptional events, most likely through loosening of chromatin structure due to the negative charge on the acetyl groups, thus facilitating access (to the transcriptional machinery) to the templates to be transcribed. While *in vitro* data from our laboratory demonstrated H4K14 to be the target site of LdHAT4 12, we were unable to confirm this to be the case *in vivo*, as the H4acetylK14 (modification-specific) antibodies we raised in rabbit (two animals were immunized), though affinity purified against unmodified H4 peptide, still cross-reacted with H4acetyK4 peptide in peptide competition assays. This indicated that it was recognizing both, H4acetylK4 and H4acetylK14 (though not unmodified H4 as it did not cross-react with the unmodified H4 peptide in peptide competition assays), thus making it impossible for us to determine if HAT4-nulls displayed an altered pattern of H4K14 acetylation, particularly in light of the fact that that H4K4 acetylation occurs in >70% H4 and H4K14 acetylation occurs only in ~1% H4 (based on results in *Trypanosoma* species). Nevertheless, we examined the effect of HAT4 deletion on global gene expression using DNA microarrays as described in Methods, comparing the transcriptome profile of HAT4-nulls with that of wild type control cells. We found that LdHAT4 knockout negatively impacted expression of only a select group of genes (Fig. 6 and Supplementary Tables S3 and S4; complete data available at GEO, Accession no: GSE76574). Moreover, the deletion of HAT4 had only a modest effect, with gene expression levels altering between 1.5-fold to 2.5 fold, perhaps in keeping with the fact that H4K14 acetylation is not a very prominent PTM in *Trypanosoma* species. In the absence of H4acetylK14 antibodies (or HAT4 antibodies) that may be used in a ChIP-based genome-wide study it is difficult to directly predict the mechanism by which this regulation is occurring. However, based on the limited number of genes whose expression is being regulated, and then too only modestly, it appears that HAT4 is not a major player in the epigenetic regulation of global gene expression in *Leishmania donovani*.

### Prolonged G2/M phase exhibited by HAT4-nulls may be due to decreased expression of Cdc20

Upon analysis of the DNA microarray data for any likely cause for the prolonged G2/M phase observed in HAT4-nulls (Fig. 3), it was observed that expression of the *cdc20* gene was down-regulated approximately 1.6-fold upon deletion of LdHAT4 (Fig. 6 and Supplementary Table S3). Cdc20 is the early mitotic activator subunit of the Anaphase Promoting Complex/Cyclosome (APC/C) that is conserved across eukaryotes and has been well studied in yeast as well as human cells. The activation of APC/C (a ubiquitin protein ligase) by Cdc20 early in mitosis is essential for smooth transitioning of cells through mitosis, as Cdc20-activated APC/C targets various proteins including the S phase cyclins and securin for ubiquitin-mediated proteasomal degradation^15^,^16^. *Leishmania donovani* 1S Cdc20 has been identified previously, and its functionality demonstrated in yeast by complementation experiments using *cdc20* conditional mutants^17^.

**Figure 7:**
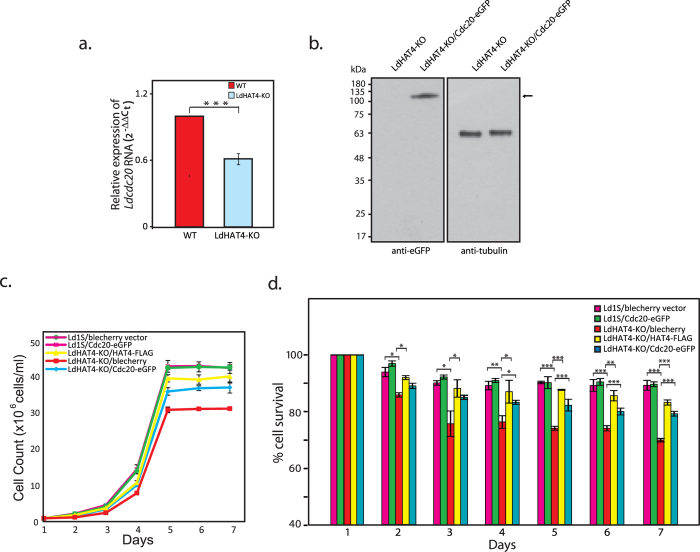
(**a**) Real Time PCR analysis. Expression of *Ldcdc20* in LdHAT4-KO cells was compared to its expression in wild type cells. Differential expression was estimated using the 2^−ΔΔC^_T_ method, as detailed in Methods. Student’s t-test (two-tailed) was applied to analyze the data. *P* value obtained: 0.0002. (**b**) Analysis of ectopic expression of LdCdc20-eGFP in LdHAT4-KO cells. Whole cell extracts from LdHAT4-KO cells and LdHAT4-KO/Cdc20-eGFP cells were analyzed by western blots using anti-eGFP antibodies (1:1000 dilution; 3.2 × 10^8^ cell equivalents loaded per cell type) and anti-tubulin antibodies (1:5000 dilution; 5 × 10^7^ cell equivalents loaded per cell type). (**c**) Growth analysis. Growth pattern of LdHAT4-KO/Cdc20-eGFP cells was compared with that of knockout line harbouring empty vector (LdHAT4-KO/blecherry), as well as with Ld1S either harbouring empty vector (Ld1S/blecherry) or expressing Cdc20-eGFP episomally (Ld1S/Cdc20-eGFP). **(d**) Analysis of cell survival. Percent LdHAT4-KO/Cdc20-eGFP survivors was compared with percent survivors in knockout line LdHAT4-KO/blecherry, as well as Ld1S/blecherry and Ld1S/Cdc20-eGFP lines, every 24 hrs over a period of seven days. For growth and survival analyses three biological replicates were initiated and carried out in parallel, and mean values are presented, with error bars indicating standard deviation. Student’s t-test (two-tailed) was applied to analyze the data. *P* values obtained: *p < 0.05, **p < 0.005, ***p < 0.0005.

To investigate if the growth and cell cycle defects of HAT4-nulls were linked to lowered expression of *cdc20* we began with confirming that *Ldcdc20* expression was downregulated in HAT4-nulls using real time PCR, as described in Methods. As seen in Fig. 7a, *Ldcdc20* RNA levels in HAT4-nulls were ~60% of the *Ldcdc20* RNA levels seen in wild type control cells, in keeping with results from the microarray analysis which showed an ~1.6 fold downregulation of *cdc20* expression in HAT4-nulls (Supplementary Table S3). To ascertain if LdHAT4 was regulating G2/M events via LdCdc20 we attempted to rescue the HAT4-null mutant phenotypes by expressing LdCdc20 ectopically in LdHAT4-KO cells. Accordingly, LdCdc20 was expressed in fusion with eGFP in LdHAT4-KO cells (cloning and transfection details in Supplementary Methods), with expression of the full-length LdCdc20-eGFP protein in HAT4-nulls (LdHAT4-KO/Cdc20-eGFP cells) being verified by western blotting using anti-eGFP antibodies (Fig. 7b) and immunofluorescence analysis revealing that the protein localized to the cytosol (data not shown), as reported earlier^17^. A comparison of the growth of LdHAT4-KO/Cdc20-eGFP cells with LdHAT4-KO cells revealed that the growth and survival defects exhibited by LdHAT4-KO cells were largely alleviated by expression of LdCdc20 in HAT4-nulls (Fig. 7c,d), though not to the same extent as ectopic expression of HAT4-FLAG in HAT4-nulls.

**Figure 8:**
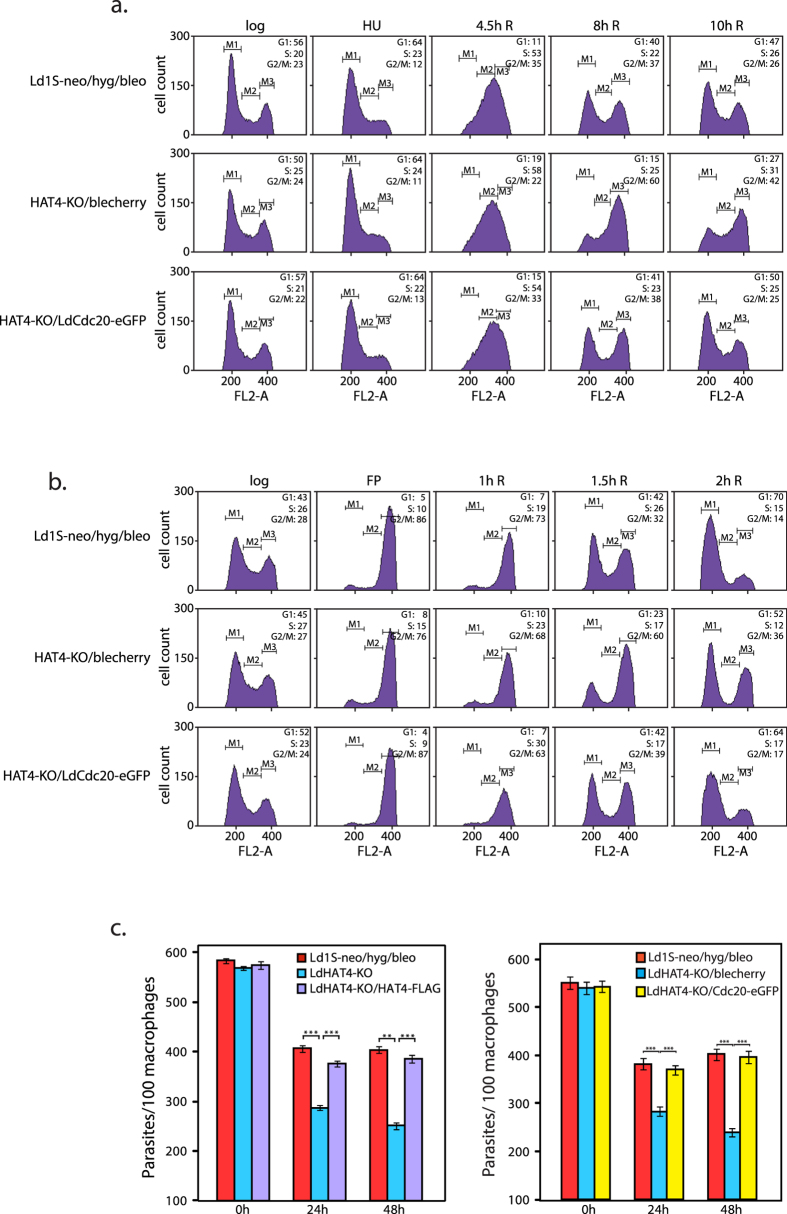
(**a,b**) Flow cytometry profiles comparing LdHAT4-KO cells expressing Cdc20-eGFP ectopically and LdHAT4-KO cells. For each cell type 30,000 events were recorded at every time-point, and data were analyzed using CellQuest Pro Software (BD Biosciences). M1, M2 and M3 gates indicate G1, S and G2/M phases. Time indicated above each histogram indicates the time after release from block. Percent of cells in G1, S and G2/M at the different time-points are indicated in the upper right-hand corner of each histogram box. (**a**) Promastigotes were synchronized at G1/S using 5 mM hydroxyurea and released into S phase. (**b**) Promastigotes were synchronized at G2/M using 5 μM flavopiridol and released into G1 phase. Both experiments were performed three times, with one dataset of each being presented here. Combined statistical analyses of all data sets are presented in Supplementary Tables 5 and 6. (**c**) Left panel: Effect of ectopic expression of HAT4-FLAG in LdHAT4-KO on parasite survival within macrophages. Right panel: Effect of ectopic expression of Cdc20-eGFP in LdHAT4-KO on parasite survival within macrophages. Three replicates of each experiment were separately initiated and carried out in parallel. The number of parasites within macrophages were scored microscopically using DAPI staining and Z-stack image analysis with the help of a confocal microscope Mean values are presented in the bar charts. Error bars indicate standard deviation. Student’s t-test (two-tailed) was applied to analyze the data. *P* values obtained: ***p < 0.0005, **p < 0.005.

LdHAT4-KO/Cdc20-eGFP cells were synchronized with hydroxyurea to compare their cell cycle profiles with HAT4-nulls harbouring empty vector. As evident from the data in Fig. 8a (and Supplementary Table S5), the ectopic expression of LdCdc20 in HAT4-nulls allowed the cells to recover from the aberrant cell cycle pattern of LdHAT4-KO cells, with cells transitioning through G2/M into G1 at rates almost comparable with control cells. The abrogation of the length of the G2/M span-period seen in HAT4-nulls by ectopic expression of LdCdc20 in these cells, was confirmed by synchronization using flavopiridol. It was observed that LdHAT4-KO/Cdc20-eGFP cells were released from the flavopiridol-induced G2/M arrest without the delay detected in case of HAT4-nulls (Fig. 8b and Supplementary Table S6). To investigate if altered levels of Cdc20 expression are also the cause of decreased survival of HAT4-nulls in macrophages, J774A.1 cells were infected with LdHAT4-KO/Cdc20-eGFP metacyclics and infection assessed microscopically as before. As seen in Fig. 8c right panel, the number of parasites per hundred macrophages in case of LdHAT4-KO/Cdc20-eGFP was comparable with parasites in which HAT4 had not been deleted (Ld1S-neo/hyg/bleo). This pattern was similar to what was observed in macrophages infected with LdHAT4-KO/HAT4-FLAG metacyclics (Fig. 8c left panel). Thus, G2/M defects appear to be a primary cause of decreased survival of HAT4-nulls in macrophages.

## Discussion

Histone acetylation events in eukaryotes play a dual role in modulating chromatin environment. On the one hand, specific acetylation events (histone H4 diacetylation at K5 and K12 residues) precede nucleosomal deposition and contribute to establishing global chromatin structure^18^, while on the other hand hyperacetylation of histones at specific residues is believed to promote the accessibility of chromatin to cellular machineries involved in DNA replication, transcription and repair, presumably due to the negative charge conferred by acetylation causing a loosening of histone-DNA contacts (reviewed in^3^). These two classes of events are governed by two different sets of histone acetyltransferases, with the cytosolic Type B HATs mediating histone acetylation events required for chromatin assembly and the nuclear Type A HATs mediating histone acetylation events that promote DNA-related cellular transactions^19^. The MYST-family of histone acetyltransferases belong to the Type A category, and are ubiquitously found across eukaryotes.

Four MYST-family HATs have been identified in *Leishmania* species, three of which (HATs1–3) are conserved across all *Trypanosoma* species as well. HATs 1–3 have been characterized in *T. brucei*, and while TbHAT2 has been identified to target H4K10 acetylation and TbHAT3 specifically targets H4K4 acetylation, the target site of TbHAT1 has not yet been identified^20^,^21^. All three TbHATs are constitutively nuclear, and of the three HATs only TbHAT1 and TbHAT2 are essential for cell survival, with TbHAT1 being implicated in the modulation of telomeric silencing^20^. While TbHAT3 was shown to be non-essential to cell survival, a later study found that it mediates locus-specific responses to double-strand breaks in DNA, thus facilitating DNA repair^22^. Our laboratory has found HAT3 to play a role in arbitrating DNA repair events in *Leishmania donovani* also, with HAT3-mediated acetylation of proliferating cell nuclear antigen (PCNA) flagging PCNA for subsequent monoubiquitination in order to facilitate translesion DNA synthesis, following UV-induced DNA damage. LdHAT3-mediated H4K4 acetylation was found to play a role in histone deposition as well, with deletion of HAT3 gene causing decreased cell viability presumably due to defects in histone deposition^13^.

The fourth trypanosomatid MYST-family HAT (HAT4) is absent in *T. brucei* (but present in *T. cruzi*), and has not been functionally characterized *in vivo* in any trypanosomatid thus far. Previous work from our laboratory identified the H4K14 residue to be the target site of LdHAT4 *in vitro*. We also found LdHAT4 to be a less typical MYST-family member in that it is mainly cytosolic in nature, being found in the cytoplasm at all stages of the cell cycle though also found in the nucleus in G2/M phase^12^. To investigate the role of LdHAT4 in regulating *Leishmania* cellular events we took the approach of creating a HAT4-null parasite (Fig.1) and analyzing its phenotypes. As apparent from our success in creating the HAT4-null, this enzyme is not essential to the parasite. However, decreased cell viability (Fig. 2a,b) indicated that HAT4 played a role in regulating one or more vital biological process in *Leishmania* promastigotes. Analysis of the distribution of histone H4 over the soluble and chromatin-bound fractions of the cell revealed that any acetylation event mediated by HAT4 does not play a role in histone deposition (Fig. 2c). Considering that HAT4 acetylates H4K14 *in vitro* and is likely to do so *in vivo* as well (though we have no evidence for it in the absence of quality modification-specific antibodies), these findings are in keeping with what was recently reported in *Trypanosoma cruzi* where histone H4 mutated at K14 was still deposited in nucleosomes^14^. We found HAT4 to exercise a modest effect on the expression of a small set of genes (Fig. 6), possibly in tune with the fact that only about 1% of histone H4 has been reported to be acetylated at K14 in *Trypanosoma* species. We also analyzed the genomic locations of the down-regulated and up-regulated genes in the light of published information regarding the genome-wide distribution of H3 acetylation and methylation, H2A.Z, H2B.V, H3V and H4V variants, and base J^7^,^8^,^23^,^24^,^25^,^26^. In cases where no information regarding *Leishmania* species is available we examined the relevant syntenic regions in the *Trypanosoma* species. However, we were unable to find any correlation between the positions of the regulated genes and the locations of these epigenetic markers. It is possible that this regulation of a select set of genes is an indirect effect of HAT4 deletion - perhaps impaired acetylation of one or more specific proteins that control expression of these genes. The behavior of LdHAT4 contrasts that of LdHAT3 and LdHAT2, with LdHAT3 playing no role in regulating gene expression and LdHAT2 modulating gene expression of a large number of genes (unpublished results from our lab), suggesting that there is minimal functional redundancy among the *Leishmania* HATs unlike what is seen in yeast and higher eukaryotes.

Flow cytometry analyses revealed that LdHAT4 modulates events occurring in G2/M stages of the cell cycle with HAT4-nulls exhibiting a longer than usual G2/M (Fig. 3). The regulation of cell cycle progression is a complex process involving a myriad of governing molecules. Some of these regulators are expressed in a stage-specific manner while others exert their effect via their interactions with other proteins that occur/are active only at a specific stage of the cell cycle, and a fine balance is maintained among various regulatory factors to promote a smooth navigation of cells through the different stages. While DNA microarray analysis of HAT4-null cells revealed that only a small set of genes were differentially regulated in these cells as compared to wild type cells (Fig. 6), *cdc20* was identified as one among the downregulated genes. Cdc20 (**c**ell **d**ivision **c**ycle 20) is one of the two main regulators of the activity of the Anaphase Promoting Complex/Cyclosome (APC/C), being conserved in eukaryotes and well studied in yeast and human cells. The APC/C is a multisubunit E3 ubiquitin protein ligase that exercises its influence from metaphase to the G1/S transition, and is activated by Cdc20 and by Cdh1 in sequential manner. The activation of the APC/C by two different regulators ensures that two different sets of substrates are targeted for degradation by APC/C in a temporal manner. Thus, APC/C^Cdc20^ targets the degradation of S and M phase cyclins, securin, and other proteins in metaphase^27^, while APC/C^Cdh1^ targets degradation of aurora kinases in anaphase, thus regulating reorganization of the mitotic spindle to promote exit from mitosis followed by cytokinesis^28^. The timely degradation of these various proteins is majorly instrumental in pushing the cells through mitosis into G1, and multiple mechanisms regulate the temporal order of target protein degradation (reviewed in^15^). A recent study by Lu *et al*.^29^ indicates that these include the multi-step nature of ubiquitination, differences in the strengths of protein-protein interactions between substrate and APC/C^Cdc20^, and competition among the target proteins. While the APC/C pathway has been extensively examined as the major mode by which Cdc20 exerts its effects, a second protein named Parkin (also a ubiquitin ligase) has been recently identified as an alternate partner through which Cdc20 may act^30^.

To investigate if G2/M defects seen in HAT4-nulls was due to lowered expression of *cdc20*, we first confirmed that *cdc20* expression is downregulated in HAT4-nulls using real time PCR (Fig. 7a). Ectopic expression of Cdc20 in HAT4-nulls was found to alleviate HAT4-null growth and cell cycle defects (Figs 7 and 8). Considering that both, DNA microarray analysis as well as real time PCR data revealed that expression of *cdc20* in HAT4-nulls is about 60% that seen in wild type cells (1.6 fold downregulation), it appears that *cdc20* expression levels must match or exceed a certain threshold for cells to smoothly transition through G2/M into G1. It was observed that while cell cycle defects vis-à-vis prolonged G2/M were relieved by Cdc20 expression ectopically, growth defects were not relieved to the same extent as by episomal expression of HAT4-FLAG. This suggests that one or more of the other genes whose expression is modulated by HAT4 is also critical for promastigote growth and survival. LdHAT4 was found to be vital not only for viability of *Leishmania* promastigotes, but also for the survival of the parasite within host macrophages (Fig. 5b). G2/M defects caused by downregulation of *cdc20* linked to HAT4 deletion are the likely cause of the observed decrease in survival, as expression of Cdc20 in HAT4-nulls surmounted parasite growth defects within macrophages (Fig. 8c). While there is no direct evidence demonstrating APC/C-mediated ubiquitin-dependent targeted degradation of proteins during mitosis in *Leishmania* species, studies from *Trypanosoma* species suggest that this pathway is operational in trypanosomatids^31^. *Leishmania donovani* Cdc20 has been previously demonstrated to be a functional protein in yeast using complementation analyses in a *S. cerevisiae cdc20* conditional mutant strain, and overexpression of LdCdc20 in *Leishmania* promastigotes accelerated the cell cycle in general, with cells moving through all the stages quicker than usual^17^. Our results demonstrate a role for LdHAT4 in modulating *Leishmania* G2/M events and favoring the smooth passage of *Leishmania* cells into G1 phase via the tight regulation of *Ldcdc20* expression. This is the first report investigating the *in vivo* role of HAT4 in any trypanosomatid, also the first study directly identifying Cdc20 as functionally important to navigation across G2/M in trypanosomatids. LdCdc20 is one of only two protozoan Cdc20 proteins to be studied thus far. *Plasmodium berghei* Cdc20 has been found to be important for male gametogenesis but non-essential to mitosis in the asexual blood stage^32^. Further studies will be directed towards attempting to identify downstream substrates that may be targeted by an APC/C^Cdc20^ pathway in *Leishmania*.

## Methods

### *Leishmania* cultures, growth analyses and synchronization regimes

*Leishmania donovani* 1S promastigotes were maintained in liquid culture as described^33^. Growth and cell viability analyses were carried out as detailed earlier^13^. Briefly, promastigote cultures of different *Leishmania* lines were initiated from stationary phase cultures, at a cell density of 1 × 10^6^ cells/ml. Live and dead cells were counted every 24 hours from withdrawn aliquots. Viability of cells was determined using the trypan blue exclusion method as detailed earlier^13^. Cell survival (%) was calculated by dividing the number of live cells (those that excluded trypan blue) by the total (live plus dead cells) followed by multiplication of the obtained value by 100. For every experiment, three biological replicates were initiated and carried out in parallel, and mean values are presented, with error bars indicating standard deviation. *Leishmania* whole cell lysates were isolated using the M-PER kit (Pierce Biotechnologies).

Synchronization of promastigotes using hydroxyurea was performed as described earlier^34^. Briefly, logarithmically growing promastigotes were incubated in complete M199 medium (Lonza) containing 5 mM hydroxyurea (HU) for 8 h, at a density of 2 × 10^7^ cells/ml. After removing cell aggregates by low speed centrifugation (230 *g*) the rest of the cells were washed with serum-free M199 and then released into drug-free complete M199 medium. Synchronization of promastigotes using flavopiridol was done according to the method of Listovsky *et al*.^17^. Briefly, logarithmically growing promastigotes were incubated in complete M199 medium containing 5 μM flavopiridol at a density of 2 × 10^7^ cells/ml for 16 hours, cells washed in serum-free M199, and released into drug-free complete M199 medium. Drugs neomycin/hygromycin/bleomycin were withdrawn from the cultures 72 hours before setting up the blocks with hydroxyurea or flavopiridol. Aliquots of cells removed at different times were fixed for flow cytometry analysis of total DNA content of cells, which was performed using propidium iodide (PI) staining as described^34^. Briefly, 2 × 10^7^ promastigotes were harvested per time-point, washed with 1X PBS, and fixed at 4 °C overnight in 70% methanol/30% PBS. After washing the fixed cells with 1X PBS cells were incubated in PBS containing RNase and PI (both at 100 μg/ml) for half an hour at 37 °C. Flow cytometry was performed with BD FACSCalibur flow cytometer, with 30,000 events being recorded at every time-point. Data analysis was carried out using the CellQuest Pro software (BD Biosciences), with gates M1, M2 and M3 on the histograms indicating G1, S and G2/M phases respectively. Every experiment was performed thrice, and for each experiment one data set has been shown as a representation of the results obtained. Combined statistical analyses of the three data sets for the different experiments are included in Supplementary Information.

### *Leishmania* transfections

Transfections were performed as described^35^. For making clonal lines transfected cells were plated on semi-solid M199 medium containing the appropriate drug 24 hours post- transfection, after removing cell clumps by centrifugation (200 *g* for 4 min). Colonies obtained (10–12 days later) were inoculated in M199 complete medium and expanded step-wise in liquid culture in the presence of the relevant drug. In making clonal lines, selection pressure was induced by addition of G418 at 50 μg ml^−1^, hygromycin at 16 μg ml^−1^, and/or bleomycin at 2.5 μg ml^−1^. All clonal lines were maintained in the presence of the specific selection drug(s).

### Creation of HAT4 knockout and rescue lines

The first allele was replaced with neomycin resistance gene, using homologous recombination with a donor cassette released from plasmid *HAT4-KO/neo* by EcoRV digestion (details of construction of donor plasmids in Supplementary Methods). The second allele was replaced with hygromycin resistance gene, by transfecting a donor cassette similarly released from plasmid *HAT4-KO/hyg* into LdHAT4-**h**eterozygous **k**n**o**ckout (LdHAT4-hKO) cells. LdHAT4-hKO clonal lines were selected for using G418 (50 μg ml^−1^) and LdHAT4-**k**nock**o**ut (LdHAT4-KO) clonal lines were selected for using G418 (50 μg ml^−1^) and hygromycin (16 μg ml^−1^). Clonal lines were screened by PCRs across the deletion junctions. The LdHAT4-KO rescue line was made by transfecting plasmid pXG(*bleo*)/HAT4-FLAG (cloning details in Supplementary Methods) into LdHAT4-KO cells and selecting for clonal lines using hygromycin (16 μg ml^−1^), G418 (50 μg ml^−1^l) and bleomycin (2.5 μg ml^−1^). Clonal lines were screened for LdHAT4-FLAG expression by Western Blot using anti-FLAG antibodies (Sigma Aldrich).

### Analysis of histone compartmentalization

Chromatin**-bound protein fractions were isolated from logarithmically growing promastigotes as detailed previously and analyzed by Western Blot using antibodies to H4acetylK4 and unmodified H4^13^. Fractions S1 and S2 represent the isolated soluble fractions while fractions S3 and S4 represent the isolated DNA-associated fractions.

### Analysis of by EdU labeling

Ld1S and LdHAT4-KO promastigotes were synchronized at G1/S using hydroxyurea as described earlier. Cells were released into S phase in drug-free medium, and at different time-points after release 4 × 10^6^ promastigotes (1–1.5 ml aliquots of cells) were pulsed with 5-ethynyl-2-deoxyuridine (EdU; 160 μM) in M199 medium for 15 min at 26 °C and then harvested. Harvested cells were washed with 1X PBS, fixed in 2% PFA, cell spreads made, and stained for EdU uptake using the Click-iT EdU Imaging Kit (Invitrogen; Cat. No. C10337) as per the manufacturer’s instructions. Stained cells were visualized by confocal microscopy. Images were captured and analyzed with a LeicaTCS SP5 confocal microscope using a 100X (in oil) objective at room temperature, using Leica LAS AF software.

### Isolation of RNA, DNA microarray analysis and RT-PCRs

Total RNA was isolated from *Leishmania* promastigotes using the PureLink RNA mini kit (Invitrogen, USA), cDNA synthesis was carried out using the iScript cDNA synthesis kit (Bio-Rad Laboratories, USA), and PCRs performed using one-tenth of the synthesized cDNA, as described earlier^13^. In knockout lines, *LdHAT4* expression was analyzed using primers HAT4-RT-F1 and HAT4-RT-F2 (5′-CCAACCTCTCCGACCTCT-3′ and 5′-TGAAGCTCTGCTGCTGCCT-3′ respectively) while *tubulin* expression was analyzed using primers Tub-RT-F1 and Tub-RT-R2 (5′-CTTCAAGTGCGGCATCAACTA-3′ and 5′-TTAGTACTCCTCGACGTCCTC-3′). Products were analyzed by agarose gel electrophoresis (1.2% agarose in 1X TBE).

DNA microarray analysis was carried out with biological replicates, using RNA isolated from logarithmically growing promastigotes (RNA was isolated from each cell type in two separate experiments using the PureLink RNA mini kit). Bioanalyzer profiles of the isolated RNA samples confirmed that the purity and integrity of the RNA were optimal for microarray analysis. One-colour microarray-based gene expression analysis was carried out after labeling complementary RNA with Cy3 using Agilent’s Quick-Amp labeling Kit, and analyzing the labeled RNA using Gene Expression *Leishmania* 8 × 15 K, AMADID: 035638 (Genotypic Technology), with the help of Agilent’s *in situ* hybridization kit. The data were analyzed using GeneSpring GX Version 12.1.

Real time PCR was performed using VeriQuest SYBR Green qPCR Master Mix (Affymetrix) according to the manufacturer’s instructions. *Ldcdc20* expression was analyzed using Cdc20-RT-F and Cdc20-RT-R primers (5′-CCTATAGCTCTAC GAAAGGCT-3′ and 5′-ATTCATGAGCGAGCGGCTG-3′ respectively), and *tubulin* expression was analyzed using Tub-RT-F1 and Tub-RT-R2. The reactions were run in the 7900HT Real Time PCR system (Applied Biosystems). Differential expression of *Ldcdc20* in HAT4-nulls versus wild type cells was estimated using the 2^−ΔΔC^_T_ method^36^. The experiment was performed thrice, and in each experiment the samples were analyzed in triplicates. In each experiment, the average C_T_ values for *Ldcdc20* and *tubulin* were determined (mean of triplicate samples, for each). The average C_T_ values were used to calculate the ΔC_T_ values (average C_T_ value for *tubulin* deducted from average C_T_ value for *Ldcdc20*). These were then used to calculate the ΔΔCT value (ΔC_T_ value for RNA isolated from wild type cells deducted from ΔC_T_ value for RNA isolated from HAT4-null cells), from which the 2^−ΔΔC^_T_ value was determined. The mean 2^−ΔΔC^_T_ value (from the three experiments) is presented in a bar chart, with error bars indicating standard deviation.

### Macrophage infection experiment

J774A.1 cells (fresh thaw) were infected with *Leishmania* metacyclics after two passages of the macrophages. The cells were cultured in DMEM (Life Technologies) supplemented with heat inactivated fetal bovine serum (10%), and maintained in an atmosphere of 5% CO_2_ at 37 °C. For infection, 1 × 10^6^ macrophages (in 2 ml complete DMEM) were plated on a poly-lysine coated coverslip placed in the well of a six-well cluster dish, 24 hours before infection. Infection was carried out by addition of 2 × 10^7^ metacyclics to the well in serum-free DMEM, and incubating the infection mix at 37 °C/5% CO_2_ for 5 hours. The medium (along with the remaining metacyclics) was then removed by aspiration, the wells (carrying infected macrophages on coverslip) rinsed twice with PBS, and 2 ml complete DMEM medium added. The cells were then further incubated for 24 hours or 48 hours at 37 °C/5% CO_2._ Macrophage infections were analyzed by imaging using a LeicaTCS SP5 confocal microscope with a 100X objective. For this, the coverslip-adhered macrophages were fixed with 4% paraformaldehyde at room temperature for 15 minutes, permeabilized with 0.25% TritonX-100 for 5 minutes, washed with PBS, and mounted in anti-fade solution containing DAPI (Vectashield, Vector Laboratories). Z-stacks were acquired to determine the number of parasites within the macrophages, using DAPI staining to score the parasites. Images were analyzed using Leica LAS AF software.

### Data availability

The complete DNA microarray data (MIAME-compliant) has been deposited at Gene Expression Omnibus: Accession number GSE76574.

## Additional Information

**How to cite this article**: Yadav, A. *et al*. Histone acetyltransferase HAT4 modulates navigation across G2/M and re-entry into G1 in *Leishmania donovani*. *Sci. Rep*. **6**, 27510; doi: 10.1038/srep27510 (2016).

## Supplementary Material

Supplementary Information
