# Supplementary material for: Histone acetyltransferase HAT4 modulates navigation across G2/M and re-entry into G1 in Leishmania donovani
Source: Sci Rep. 2016 Jun 8;6:27510. doi: 10.1038/srep27510 (PMC4897741; doi:10.1038/srep27510)
Supplement: Supplementary Information [file srep27510-s1.pdf]

Histone acetyltransferase HAT4 modulates navigation across G2/M and re-entry into G1 in *Leishmania donovani*

Aarti Yadav, Udita Chandra & Swati Saha*

Department of Microbiology, University of Delhi South Campus, New Delhi-110021, India.

Running Head: Functional role of *Leishmania* histone acetylase HAT4

*Address correspondence to Swati Saha, ss5gp@yahoo.co.in

Keywords: *Leishmania,* histone acetyltransferase, MYST family, chromatin, histone modification, trypanosomatid, Cdc20

Supplementary Methods:

Construction of knockout and rescue plasmids

Knockout constructs were made using two backbone vectors – pLEXSY_I-egfp-neo3 (purchased from Jena Biosciences, Germany), and pLEXSY-eGFP-hyg that was made as part of this study. To make pLEXSY-eGFP-hyg, the *hyg*^{r} cassette was amplified from pUC/*hygro* template[13] using primers Hyg-F/pLEXSY-eGFP and Hyg-R/pLEXSY-eGFP (5'-TCGGATCCAATATGAAAAAGCCTGAACTC-3' and 5'-TCACTAGTCTACTC TATTCCTTTGCCCT-3'), and the amplicon cloned into the BamHI-SpeI sites of plasmid pLEXSY_I-egfp-neo3, thus replacing the *neo*^{r} cassette with the *hyg*^{r} cassette.

The 5' and 3' flank sequences of HAT4 were amplified off genomic DNA using suitable primers (HAT4-5'FL-F and HAT4-5'FL-R for 5' flank sequence: 5'-TCGCGGCCGC**GATATC**TCCACTGTGTGCCCGCACGCT-3' and 5'-TCGCGGCCGC CGTGAAGGCCCCACCCCTGCGCT-3' respectively; and HAT4-3'FL-F and HAT4-3'FL-R for 3' flank sequence: 5'-TCACTAGTACAGTATTCATGGAGGTGCAGAGAGG-3' and 5'-TCACTAGT**GATATC**CACACCCGCACACGTACGT- 3' respectively). The 5' flank sequence was cloned into the NotI site of both vectors (pLEXSY_I-egfp-neo3 and pLEXSY-eGFP-hyg), and the 3' flank sequence was cloned into the SpeI site of the two 5' flank clones generated. The orientation of inserts in all clones were checked with appropriate restriction digestion, and donor cassettes for homologous recombination were released from the two donor plasmids *HAT4-KO/neo* and *HAT4-KO/hyg* using EcoRV digestion (as indicated in bold lettering in primer sequences above). The donor cassette DNA fragments were gel purified prior to transfections for homologous recombination.

To construct the rescue plasmid pXG(*bleo*)/HAT4-FLAG, the LdHAT4 gene was transferred from plasmid pXG/HAT4-GFP[12] to pXG*(bleo)/*FLAG[13] using BamHI-EcoRV sites.

Cloning of Ldcdc20 gene and expression in Leishmania promastigotes

The gene encoding LdCdc20 was amplified using genomic DNA template and primers LdCdc20-F and LdCdc20-R (5'- CACC**CCATGG**CCACCATGGAGTTCAGAACAC-3' and 5'- A**CCATGG**CTCCTCGCAGCTCAATCTCT - 3'), and the amplicon cloned into the NcoI site of pLEXSY_I-egfp-neo3 in order to express LdCdc20 in fusion with eGFP at its C-terminus. The neomycin resistance cassette in this clone (pLEXSY-LdCdc20-eGFP/neo was then replaced with a blecherry cassette (released from the plasmid pLEXSY_I-blecherry3 by BamHI-SpeI restriction digestion and cloned into BamHI-SpeI sites of pLEXSY-LdCdc20-eGFP/neo) to create the plasmid pLEXSY-LdCdc20-eGFP/blecherry. While the pLEXSY_I-egfp vectors are designed to be tet-inducible vectors, experiments in our laboratory with six different proteins have found that the promoter in case of pLEXSY_I-egfp-neo3 supports the constitutive expression of eGFP fusion proteins in *Leishmania donovani* 1S (based on western blots as well as immunofluorescence analysis), and thus we have been using this vector for constitutive ectopic expression of proteins. The plasmid pLEXSY-LdCdc20-eGFP/blecherry was transfected into LdHAT4-KO cells and clonals selected for using all three drugs (neomycin, hygromycin and bleomycin). LdCdc20 expression in clonals was analyzed by microscopy, and a clonal showing expression in more than 90% cells was used for further analysis.

Supplementary Data

Table S1: Flow cytometry analysis of HU-synchronized HAT4-nulls

Timepoints		Ld1S-neo/hyg	LdHAT4-KO
log	G1	48.34±0.76	47.04±0.67
	S	23.30±1.23	24.09±0.07
	G2/M	26.54±0.30	25.81±0.72
HU	G1	66.45±1.32	65.08±1.54
	S	17.66±1.27	20.86±1.48
	G2/M	13.82±1.04	12.89±1.23
3h R	G1	13.86±0.47	18.74±1.85
	S	62.83±1.43	61.96±1.53
	G2/M	21.38±0.91	18.24±1.01
4.5h R	G1	9.68±0.95	13.53±1.06
	S	49.16±2.32	56.25±3.75
	G2/M	40.29±3.74	29.66±3.74
6.5h R	G1	22.20±0.43	13.25±1.15
	S	24.42±4.32	37.04±2.04
	G2/M	51.16±3.77	47.49±2.48
10h R	G1	40.11*	18.8*
	S	23.38*	28.62*
	G2/M	34.21*	47.66*
12h R	G1	42.44*	25.77*
	S	24.06*	29.40*
	G2/M	30.84*	41.12*

Three independent experiments were carried out. In every experiment, for each time-point 30,000 events were recorded per cell line. Numbers represent percent of analyzed cells (mean of three experiments) and numbers following ± sign represent s.e.m. values. * indicates mean of two experiments. HU: hydroxyurea.

Table S2: Flow cytometry analysis of FP-synchronized HAT4-nulls

**Timepoints**		**Ld1S-neo/hyg**	**LdHAT4-KO**
log	G1	50.02±0.45	46.01±0.16
	S	21.19±0.74	21.94±0.58
	G2/M	25.95±1.25	29.79±0.75
FP	G1	12.68±1.28	8.82±0.73
	S	10.58±0.61	11.02±0.45
	G2/M	75.50±0.80	77.99±1.95
0.5h R	G1	14.32±1.50	10.95±0.60
	S	14.92±1.76	18.22±1.34
	G2/M	68.31±0.39	70.90±2.29
1.5h R	G1	37.10±1.76	20.04±3.51
	S	9.39±0.63	18.45±3.83
	G2/M	49.64±3.62	61.01±0.71
2h R	G1	68.14±3.06	59.37±4.85
	S	18.7±2.07	7.97±0.77
	G2/M	10.51±0.95	30.59±3.10

Three independent experiments were carried out. In every experiment, for each time-point 30,000 events were recorded per cell line. Numbers represent percent of analyzed cells (mean of three experiments) and numbers following ± sign represent s.e.m. values. FP: Flavopiridol.

Table S3: Downregulated genes in HAT4-nulls

Probe Name/ Gene number	P value	Fold Change experiment 1	Fold Change experiment 2	Average Fold Change	Regulation	Description
LDBPK_220500	0.0132	5.0281	4.8905	4.9593	Down	Hypothetical protein
LDBPK_320110	0.0009	2.0705	2.1435	2.1070	Down	Mitochondrial carrier protein
LDBPK_322590	0.0032	2.0139	2.1885	2.1012	Down	Mitochondrial RNA binding complex 1 subunit
LDBPK_272330	0.0967	1.815	2.2815	2.0483	Down	Hypothetical protein
LDBPK_343610	0.0440	2.042	1.8531	1.9476	Down	Hypothetical protein
LDBPK_070070	0.0919	1.7411	2.0279	1.8845	Down	Hypothetical protein
LDBPK_020170	0.0123	1.8921	1.8661	1.8791	Down	Phosphoglycan beta 1,3 galactosyltransferase
LDBPK_333350	0.1975	1.6471	2.0000	1.8236	Down	Cation transporter
LDBPK_201240	0.0697	2.0279	1.5368	1.7824	Down	Calpain-like cysteine peptidase
LDBPK_282140	0.1783	1.591	1.9453	1.7682	Down	Protein kinase
LDBPK_010380	0.1392	1.5476	1.9052	1.7264	Down	Hypothetical protein
LDBPK_020490	0.1031	1.8661	1.5801	1.7231	Down	Hypothetical protein
LDBPK_250760	0.0092	1.6586	1.729	1.6939	Down	Eukaryotic initiation factor 5a
LDBPK_343170	0.0488	1.8276	1.5368	1.6823	Down	Hypothetical protein
LDBPK_211620	0.0028	1.6358	1.6817	1.6588	Down	Hypothetical protein
LDBPK_170650	0.0082	1.6245	1.6817	1.6531	Down	Hypothetical protein
LDBPK_322820	0.0336	1.4948	1.8025	1.6487	Down	Hypothetical protein
LDBPK_161140	0.0423	1.6471	1.6471	1.6472	Down	Hypothetical protein
LDBPK_352310	0.0013	1.6471	1.6471	1.6472	Down	ATP-grasp domain containing protein
LDBPK_291230	0.0083	1.5583	1.6934	1.6259	Down	Tryparedoxin-like protein
LDBPK_090760	0.0052	1.5691	1.6702	1.6197	Down	Hypothetical protein
LDBPK_351020	0.0577	1.4339	1.8025	1.6182	Down	Casein kinase I
LDBPK_352870	0.0436	1.6471	1.58	1.6136	Down	Major facilitator superfamily
LDBPK_241790	0.0256	1.7171	1.4948	1.6060	Down	Cell division cycle protein 20
LDBPK_131140	0.0431	1.4641	1.7291	1.5966	Down	Thiamin pyrophosphokinase
LDBPK_341720	0.0650	1.464	1.7171	1.5906	Down	Amastin-like surface protein
LDBPK_321400	0.0273	1.4539	1.6934	1.5737	Down	Cleavage and polyadenylation specificity factor-like protein
LDBPK_230370	0.1138	1.4439	1.6934	1.5687	Down	Hypothetical protein
LDBPK_281570	0.2509	1.6934	1.4439	1.5687	Down	Hypothetical protein
LDBPK_110520	0.0365	1.4948	1.6132	1.5541	Down	Nucleobase transporter
LDBPK_333370	0.2003	1.4142	1.6586	1.5364	Down	Hypothetical protein
LDBPK_350380	0.1279	1.4339	1.6358	1.5349	Down	Thioredoxin
LDBPK_320290	0.0038	1.4948	1.5691	1.5320	Down	B9 domain containing protein 1
LDBPK_210190	0.0280	1.6021	1.4339	1.5180	Down	Serine/threonine protein kinase

Table S4: Upregulated genes in HAT4-nulls

Probe Name/ Gene number	P value	Fold Change experiment 1	Fold Change experiment 2	Average Fold Change	Regulation	Description
LDBPK_060910	0.0019	3.9708	3.7914	3.8811	Up	Acyl-coenzyme a dehydrogenase
LDBPK_060870	0.0019	3.5122	3.8839	3.6981	Up	Hypothetical protein
LDBPK_060880	0.0034	3.432	3.7994	3.6157	Up	Hypothetical protein
LDBPK_060890	0.0004	3.385	3.4045	3.3948	Up	Dihydrofolate reductase-thymidylate synthase
LDBPK_060900	0.0011	3.2403	3.4812	3.3608	Up	Arginine N-methyltransferase type III- PRMT7
LDBPK_060860	0.0015	3.1912	3.4392	3.3152	Up	Lipin
LDBPK_010600	0.0003	2.8284	2.9105	2.8695	Up	Hypothetical protein
LDBPK_180180	0.3644	1.9675	1.8255	1.8965	Up	Hypothetical protein
LDBPK_201420	0.0020	1.778	1.7044	1.7412	Up	Pumilio protein 9
LDBPK_262260	0.0103	1.6794	1.7794	1.7294	Up	Hypothetical protein
LDBPK_354910	0.0031	1.5845	1.5272	1.5559	Up	Proteasome alpha 1 subunit
LDBPK_180190	0.0204	1.5003	1.5856	1.5430	Up	Hypothetical protein
LDBPK_364440	0.2276	1.552	1.5096	1.5308	Up	Hypothetical protein
LDBPK_181670	0.0205	1.5557	1.5055	1.5306	Up	Hypothetical protein
LDBPK_313300	0.0448	1.5353	1.5142	1.5248	Up	Protein kinase

Table S5: Flow cytometry analysis of HU-synchronized HAT4-nulls expressing LdCdc20-eGFP ectopically

**Time-points**		**Ld1S-neo/hyg/bleo**	**LdHAT4-KO/blecherry**	**LdHAT4-KO/Cdc20-eGFP**
HU	G1	65.14±2.26	64.87±0.82	67.21±1.88
	S	21.15±1.59	22.18±1.10	18.74±2.09
	G2/M	12.67±0.95	12.46±0.91	13.36±0.91
4.5h R	G1	11.19*	14.88*	16.32*
	S	51.12*	57.48*	52.09*
	G2/M	37.62*	27.68*	32.60*
8h R	G1	41.41±1.13	16.27±1.01	39.35±0.90
	S	22.08±1.07	23.99±0.55	23.79±0.71
	G2/M	35.86±2.20	60±1.31	37.05±1.34
10h R	G1	46.22±0.64	24.51±1.07	47.79±1.76
	S	21.80±2.82	30.61±0.32	21.18±2.51
	G2/M	31.47±2.97	44.92±1.53	31±2.87

Three independent experiments were carried out. In every experiment, for each time-point 30,000 events were recorded per cell line. Numbers represent percent of analyzed cells (mean of three experiments) and numbers following ± sign represent s.e.m. values. * indicates mean of two experiments. HU: hydroxyurea.

Table S6: Flow cytometry analysis of FP-synchronized HAT4-nulls expressing LdCdc20-eGFP ectopically

**Timepoints**		**Ld1S-neo/hyg/bleo**	**LdHAT4-KO/blecherry**	**LdHAT4-KO/Cdc20-eGFP**
FP	G1	5.85±0.60	6.44±1.39	3.80±0.36
	S	9.59±0.17	10.28±2.71	7.37±1.20
	G2/M	84.78±0.54	83.31±3.88	89.11±0.99
1h R	G1	7.22±0.12	8.36±1.28	5.72±0.65
	S	16.56±2.12	15.16±4.16	19.87±5.20
	G2/M	76.36±2.13	76.58±5.45	74.83±5.80
1.5h R	G1	41.65*	26.62*	40.5*
	S	17.66*	14.50*	12.85*
	G2/M	40.61*	58.71*	46.3*
2h R	G1	71.99*	50.01*	67.86*
	S	16.14*	11.52*	16.54*
	G2/M	10.76*	38.41*	13.74*

Three independent experiments were carried out. In every experiment, for each time-point 30,000 events were recorded per cell line. Numbers represent percent of analyzed cells (mean of three experiments) and numbers following ± sign represent s.e.m. values. * indicates mean of two experiments. FP: flavopiridol.
